# The continuing impact of COVID-19 on eating disorder early intervention services in England: An investigation of referral numbers and presentation characteristics

**DOI:** 10.1192/j.eurpsy.2025.10038

**Published:** 2025-05-30

**Authors:** Lucy Gallagher, Lucy Hyam, Karina Allen, Ulrike Schmidt

**Affiliations:** 1 Centre for Research in Eating and Weight Disorders, Department of Psychological Medicine, Institute of Psychiatry, Psychology and Neuroscience, King’s College London, London, UK; 2 Eating Disorders Outpatient Service, South London and Maudsley NHS Foundation Trust, London, UK

**Keywords:** COVID-19, eating disorder services, early intervention, feeding and eating disorders, national health services

## Abstract

**Background:**

The COVID-19 pandemic increased the incidence and burden of eating disorders (EDs) globally. First Episode Rapid Early Intervention for EDs (FREED) is a nationally implemented early intervention service model for young people (16–25 years) with EDs in England. This study evaluates the longer-term impact of the pandemic on presentations and service provision in FREED.

**Methods:**

Data from January 2019 to September 2024 were analyzed, including three services with pre-, pandemic, and post-pandemic data (Sample 1), and 62 services with post-pandemic data (Sample 2), 32 of which also contributed data during the pandemic. Linear mixed models examined referral numbers, duration of untreated ED, diagnostic mix, average body mass index for anorexia nervosa, and wait times.

**Results:**

In the three services with pre-, pandemic, and post-pandemic data, referrals remained significantly higher post-pandemic compared to the pre-pandemic period. Across all services, post-pandemic referrals declined compared to the pandemic period. Consistently, anorexia nervosa diagnoses decreased, and the duration of untreated ED at presentation increased post-pandemic.

**Conclusions:**

The COVID-19 pandemic has had lasting impacts on ED service provision in England. Sustained investment and national support are essential to ensure FREED services continue to meet the needs of young people with recent-onset EDs and to reduce the duration of untreated ED.

## Introduction

Eating disorders (EDs), including anorexia nervosa (AN), bulimia nervosa (BN), and binge eating disorder (BED), are severe mental disorders [1]. They are associated with multiple psychiatric and physical health complications, making them a significant public health concern [2]. During the COVID-19 pandemic, international evidence indicated major increases in the incidence of and detriment caused by EDs [3]. This included a rise in ED symptoms in the general population, a rise in new cases presenting for treatment across all service levels, worsening clinical presentations, increased relapse rates, and higher levels of distress and severity, evidenced by increased rates of emergency department visits and hospitalizations [4–7].

Since the pandemic, international evidence has highlighted important trends in ED incidence and service demand. Evidence from Germany [8] and the United States [9] indicate that hospitalizations for EDs have remained substantially elevated in the post-pandemic period. Meanwhile, data from Denmark show that diagnosed ED rates increased during the pandemic and have remained high among emerging adults [10]. The greatest rise in diagnostic incidence during COVID-19, both at the primary care level [11] and across all service levels [12], seems to have been in young females with AN aged 10–19. This age range spans adolescence to emerging adulthood, a key life stage marked by significant social, psychological, vocational, and brain development [13]. Effective early intervention in this age range is essential to facilitate full recovery and prevent long-term harm [14]. This is particularly important given the increasing incidence in this demographic, as disruptions during this transitional phase can have lasting consequences [15].

First Episode Rapid Early Intervention for Eating Disorders (FREED) is a leading early intervention model for EDs that has been implemented across the National Health Service (NHS) in England [16]. FREED has also been adopted in Canada (FREEDCan), Australia (EmergED), and the Netherlands (Vibes). FREED aims to deliver rapid access to evidence-based treatment that is tailored to both the young person’s developmental stage (emerging adulthood; ages 16–25) and the early illness stage (duration of untreated ED [DUED] of three years or less). DUED refers to the length of time between the onset of clinically significant ED symptoms (i.e., symptoms that would result in an ED diagnosis) and the initiation of evidence-based treatment. The FREED service model and pathway are described in-depth elsewhere [17]. Much of the initial evidence supporting FREED was gathered in four specialist ED services [18, 19]. The national roll-out across England started in early 2020, coinciding with the start of the COVID-19 pandemic, and was completed in March 2023. FREED is now operating in 54 NHS trusts across England, encompassing 64 distinct service localities.

To assess the impact of the COVID-19 pandemic on the delivery of FREED, we previously examined data from three services where pre- to post-pandemic comparator data were available. Between 2019 and 2021, referrals to these services rose by ~50% from the end of the first national lockdown. Furthermore, there was an increase in clinical presentations of AN, with severity comparable to pre-pandemic cases [20]. Based on these findings, we emphasized the importance of continuing to evaluate FREED to monitor the longer-term impacts of the pandemic.

The present study builds directly on our earlier work (20) but differs in two key ways. First, it extends the previous analysis – originally covering data from 2019 to 2021 – through to 2024, enabling the assessment of longer-term impacts of the COVID-19 pandemic. Second, it uses a substantially larger sample, comprising data from 9237 (compared to 502) patients across a wider range of FREED services. While there is partial overlap in the data used (i.e., data from 2019 to 2021 are included again), the expanded service coverage and extended duration provide a national-level view of trends in referrals, diagnostic composition, illness severity, and DUED over the 5 years following the COVID-19 pandemic. In addition, with the larger data set, we have been able to use more advanced statistical analyses. In addition to informing service planning, this broader analysis provides valuable insight into changing patterns of early-stage ED presentations, the lasting impact of COVID-19 on access to care (e.g., via DUED data), and considerations for the ongoing development and adaptation of early intervention services at a national level.

## Methods

### Design and sample

A repeated cross-sectional design was used to evaluate routinely collected clinical outcome data from FREED patients. The data used in this analysis are part of the FREED-4-All national dataset, covering January 2019 to September 2024. Details regarding FREED data collection procedures can be found elsewhere [21]. The FREED-4-All data are collected via a data sharing agreement between South London and Maudsley NHS Foundation Trust and participating FREED services. Patients are informed of their right to opt out of data sharing through a privacy notice and information sheet. Data are de-identified by participating services before sharing and no protected characteristics are collected, meaning that limited demographic information is available (i.e., no data on ethnicity or gender identity are available). Patients screened as eligible for FREED are retained in the dataset, regardless of whether they undergo assessment or begin treatment.

Services joined the FREED network at different points during the study period, resulting in varying data availability. Three services had data spanning the pre-pandemic, pandemic, and post-pandemic periods – these services form Sample 1 and have been analyzed in our previous work (20). An additional 31 services joined during the pandemic period. All services with available post-pandemic data were included in Sample 2, which comprises 62 services in total. Sample 2 is cumulative and includes the three original services as well as those that joined during or after the pandemic.

### Outcomes and analysis

The outcomes of interest across all services included: the total number of FREED referrals each month, the average DUED (in months) for referrals each month, the percentage of monthly referrals subsequently diagnosed with AN relative to all other diagnoses, and the average wait times from referral to offered assessment and treatment slots. The focus on the proportion of AN diagnoses relative to other diagnoses was chosen to enable direct comparison with our previous study, and because AN typically brings greater physical health risks and associated need for more intensive treatment (20). Monitoring the proportion of AN cases over time therefore provides a useful proxy for understanding the nature of clinical demand across the FREED network.

Mean scores from the Eating Disorder Examination Questionnaire (EDE-Q; [22]) and the Clinical Outcomes in Routine Evaluation-10/Outcome Measure (CORE-10/OM; [23]) were used to evaluate baseline ED and general psychopathology, where available. The average baseline body mass index (BMI; kg/m^2^) of AN patients referred each month was also assessed. BMI was assessed only for patients with AN, as it serves as a key indicator of illness severity/outcome in this group, whereas it is not a reliable severity marker/outcome for other ED diagnoses.

Given the unequal number of FREED services between the pre-pandemic and post-pandemic periods, we performed two analyses. The first analysis focused exclusively on the three services previously investigated, i.e., with data spanning the pre-pandemic to post-pandemic period (sample 1). The second analysis evaluated outcomes from the pandemic to post-pandemic periods for the whole network of services that joined later and/or that lacked pre-pandemic data (sample 2). This approach enabled us to include all available data across the two analyses while avoiding direct comparisons between the 3 services with pre-pandemic data and the 62 services with post-pandemic data.

For both analyses, scatter plots were used to descriptively assess patterns and variation over time. To determine statistically significant differences in outcomes across the three pandemic periods, linear mixed models (LMMs) were used, with time modeled as a categorical variable based on pandemic periods. The pandemic periods are defined according to the UK government [24]: pre-pandemic (January 2019–February 2020), pandemic (March 2020–September 2021), and post-pandemic (October 2021–September 2024). To account for the clustered nature of the data, service was included as a random effect in the models. Monthly referrals, DUED, and wait times were log-transformed to meet the assumptions of the LMM. For the EDE-Q and CORE-10/CORE-OM models in the three services with complete data, service was excluded due to insufficient observations to estimate the random effect variance meaningfully. For these, linear models were used instead. Model performance was assessed using *R* ^2^ values, which are detailed in Supplementary Material, Appendix 2. Subsequently, contrasts were used to compare specific pandemic periods directly. For the analysis of all services, there remains some imbalance in the number of services that joined during and after the pandemic; therefore, bootstrapping was used to quantify the uncertainty of the significant estimates provided by the LMM. All analyses were conducted in R [25] using the lmerTest packages [26].

## Results

Table 1 presents the baseline demographic and clinical characteristics of the patients included for each pandemic period.

**Table 1.** Cumulative summary of FREED patient characteristics at baseline within each period 2019–2024

	Pre-pandemic	During pandemic	Post-pandemic
**Number of services**	3	34	62
**Number of FREED patients**	141	2061	9237
**Mean age at referral (years) (SD)**	19.65 (2.51)	18.34 (2.11)	19.23 (2.35)
**Mean DUED (months) (SD)**	15.37 (9.42)	14.42 (8.92)	16.70 (9.92)
**Mean EDE-Q at referral (SD)**	3.95 (1.27)	4.16 (1.21)	4.02 (1.26)
**Mean CORE–10/OM at referral (SD)**	1.75 (0.91)	1.97 (0.74)	2.06 (0.79)
**Diagnosis % (*n*)**			
*Anorexia nervosa*	34.75 (49)	37.07 (764)	27.17 (2510)
*Avoidant/restrictive food intake disorder*	1.42 (2)	1.80 (37)	1.46 (135)
*Bulimia nervosa*	19.86 (28)	14.99 (309)	9.84 (909)
*Binge eating disorder*	2.84 (4)	3.01 (62)	2.38 (220)
*Other specified feeding or eating disorder*	18.44 (26)	9.61 (198)	8.43 (779)
*Diagnosis not known*	22.7 (32)	33.5 (691)	50.71 (4684)
**Mean BMI at referral for AN patients only (SD)**	17.74 (2.57)	17.66 (2.47)	17.61 (2.38)
**Data missingness % (*n*)**			
*DUED*	38.3 (54)	33.48 (690)	53.12 (4907)
*BMI at referral*	12.06 (17)	48.67 (1003)	54.44 (5029)
*EDE-Q at referral*	24.82 (35)	57.01 (1175)	65.06 (6010)
*CORE–10/OM at referral*	31.91 (45)	68.95 (1421)	79.68 (7360)

Abbreviations: AN, Anorexia nervosa; BMI, Body Mass Index (BMI; kg/m^2^); CORE-10/OM, Clinical Outcomes in Routine Evaluation-10/Outcome Measure; EDE-Q, Eating Disorder Examination Questionnaire; DUED, Duration of an untreated eating disorder, FREED, First Episode Rapid Early Intervention for Eating Disorders.

*Note:* All statistics are calculated cumulatively across the pandemic periods. However, two services that provided data during the pandemic period did not contribute post-pandemic data and are therefore missing from the post-pandemic column.

### Referral numbers

#### Sample 1

**Figure 1.**
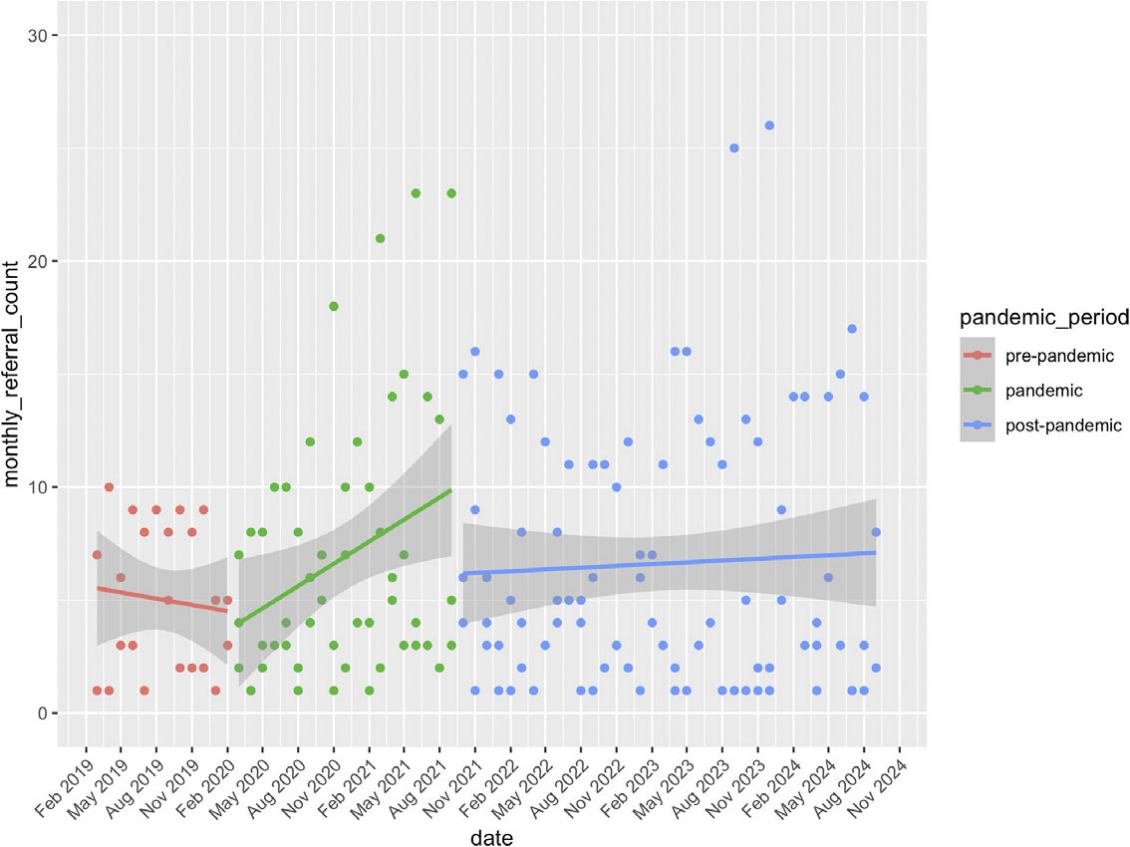
Monthly referral numbers for three services providing First Episode Rapid Early Intervention for Eating Disorders (FREED) from the pre-pandemic to post-pandemic period (January 2019–September 2024*).*

Figure 1 shows monthly referrals for the three services that have data spanning the pre-pandemic, pandemic, and post-pandemic periods. During the pandemic period, referrals increased significantly until September 2021, with an estimated increase of 51% (exp(*β*) = 1.51), SE = 0.10, *t*(167.11) = 4.13, *p* < 0.001). There was no significant difference between the pandemic and post-pandemic periods, (*β* = −0.14, SE = 0.07, *z* = −2.00, *p* = 0.11), although descriptively, there was a reduction in referrals from September 2021 to November 2021. Referrals in the post-pandemic period then remained significantly higher than pre-pandemic levels, with an estimated increase of 31% (exp(*β*) = 1.31, SE = 0.09, *t*(167.07) = 2.94, *p* = 0.004).

#### Sample 2

**Figure 2.**
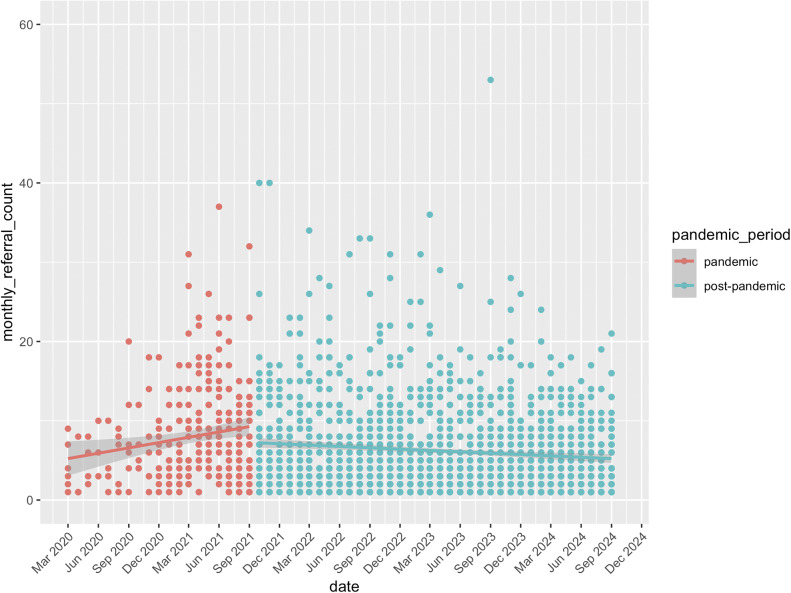
Monthly referral numbers for all services providing First Episode Rapid Early Intervention for Eating Disorders (FREED) from the pandemic to post-pandemic period (March 2020 to September 2024). Not all services provided data at all timepoints.

Figure 2 presents monthly referrals for all services with available post-pandemic data. Descriptively, a similar pattern emerges, although here, monthly referrals declined significantly in the post-pandemic period compared to the pandemic period (*β* = −0.081, SE = 0.033, *t*(1739.98) = −2.44, *p* = 0.015). This 8.1% decline was further supported by a bootstrap analysis (exp(*β*) = 0.92, 95% CI [0.86, 0.98]).

### Diagnostic mix

#### Sample 1

**Figure 3.**
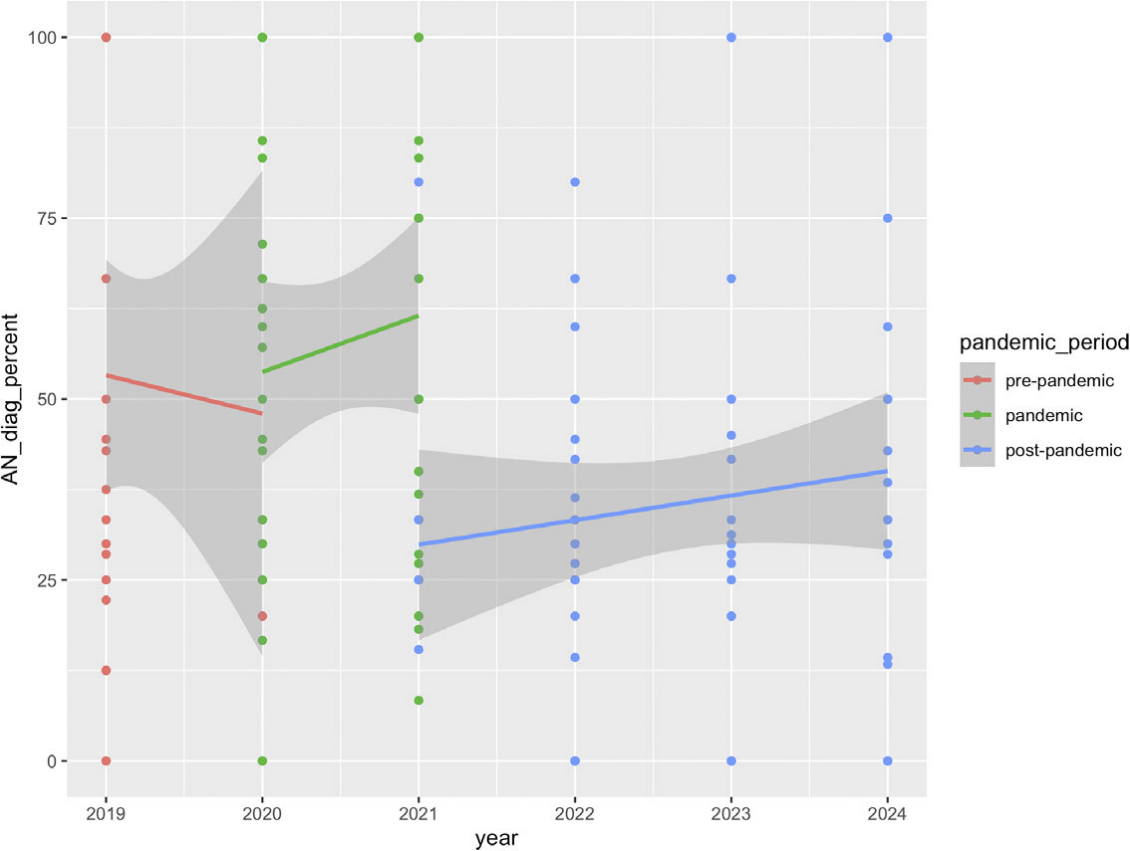
Percentage of AN diagnoses relative to other diagnoses in three services providing First Episode Rapid Early Intervention for Eating Disorders (FREED) from the pre-pandemic to post-pandemic period (January 2019–September 2024).

Figure 3 shows the percentage of AN diagnoses relative to other diagnoses for services with pre-post-pandemic data. As documented previously (Hyam et al., 2023), there was a significant increase in AN diagnosis during the pandemic (*β* = 0.63, SE = 0.17, *z* = 3.78, *p* < 0.001). AN diagnoses then significantly decreased in the post-pandemic period, compared to during the pandemic (*β* = −0.47, SE = 0.11, *z* = −4.12, *p* < 0.001), and there was no significant difference in the percentage of AN diagnoses between the pre- and post-pandemic periods (*β* = 0.16, SE = 0.16, *z* = 0.99, *p* = 0.32).

#### Sample 2

**Figure 4.**
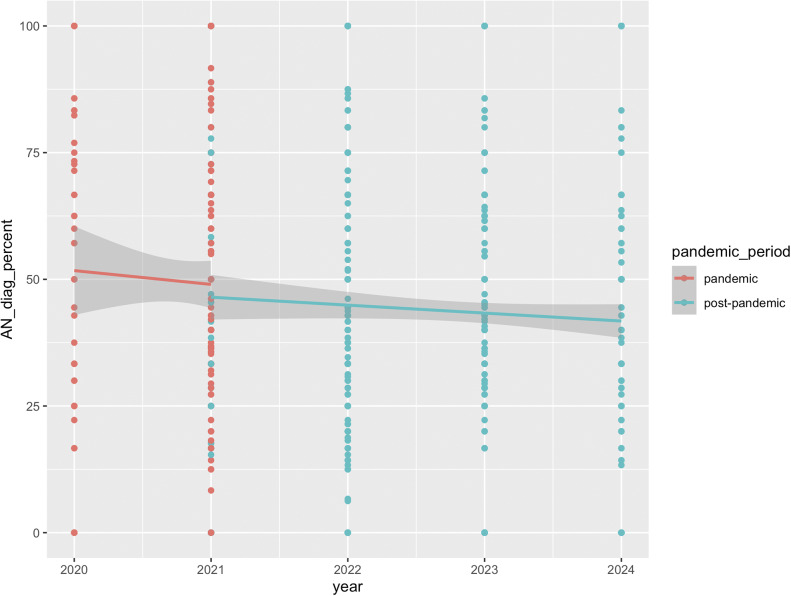
Percentage of AN diagnoses relative to other diagnoses in all services providing First Episode Rapid Early Intervention for Eating Disorders (FREED) from the pandemic to post-pandemic period (March 2020 to September 2024).

Figure 4 shows the percentage of AN diagnoses relative to other diagnoses for all services with available post-pandemic data. Like in the smaller sample, AN diagnoses significantly decreased in the post-pandemic period, compared to during the pandemic (*β* = −0.28, SE = 0.04, *z* = −6.30, *p* < 0.001). Bootstrapped results support this, with a similar mean estimate (*β* = −0.28, SE = 0.05, 95% CI [−0.38, −0.17]).

### DUED

#### Sample 1

**Figure 5.**
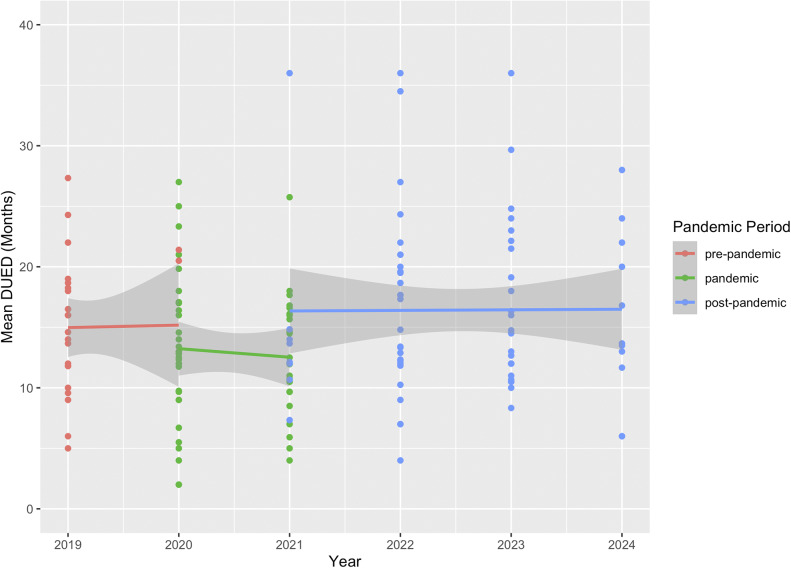
Average monthly duration of an untreated ED in three services providing First Episode Rapid Early Intervention for Eating Disorders (FREED) from the pre-pandemic to post-pandemic period (January 2019–September 2024).

Figure 5 illustrates the average DUED for the three services with complete data across all pandemic periods. There were no significant differences in the average DUED when comparing the pandemic and post-pandemic periods to the pre-pandemic period. However, the post-pandemic period showed a significant increase in the average DUED compared to the pandemic period (*β* = 0.20, SE = 0.08, *z* = 2.45, *p* < 0.05).

#### Sample 2

**Figure 6.**
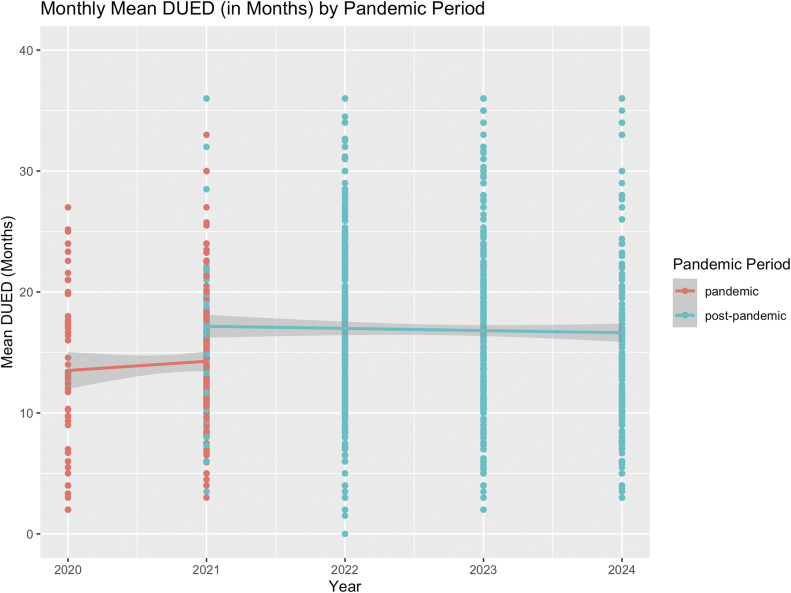
Average DUED in all services providing First Episode Rapid Early Intervention for Eating Disorders (FREED) from the pandemic to post-pandemic period (March 2020 to September 2024*).*

Figure 6 shows the average DUED for all services with available post-pandemic data. Like in the smaller sample, average DUED significantly increased in the post-pandemic period, compared to during the pandemic (*β* = 0.18, SE = 0.04, *z* = 5.09, *p* < 0.001). Bootstrapped results support this finding (*β* = 0.18, SE = 0.03, 95% CI [0.12, 0.25]).

### BMI

No significant differences were observed in the average baseline BMI of patients with AN across the pre-pandemic, pandemic, and post-pandemic periods, both for the three services with complete data spanning all periods and for all services (see Table 1).

### EDE-Q and CORE-10/OM

There were no significant differences in the average baseline EDE-Q or CORE-10/OM scores of patients across the pre-pandemic, pandemic, and post-pandemic periods, either for the three services with data spanning all periods or for all services combined (Table 1).

### Wait times

For the three services spanning all pandemic periods, assessment wait times increased by ~40% during the pandemic but returned to pre-pandemic levels post-pandemic. Treatment wait times rose by ~49% during the pandemic and remained high thereafter (Supplementary Material, Appendix 1, S1). Across all services, no significant differences in assessment or treatment wait times were observed between the pandemic and post-pandemic periods (Supplementary Material, Appendix 1, S2).

## Discussion

This study evaluated the lasting impact of the COVID-19 pandemic on FREED patient referrals and service outcomes. For the three services with pre-, pandemic, and post-pandemic data, referrals remained significantly higher post-pandemic compared to pre-pandemic levels, with no significant decline compared to the pandemic period (Figure 1). However, when analyzing all services with post-pandemic data, a significant decline in referrals was observed in the post-pandemic period compared to the pandemic period (Figure 2). This divergence reflects the variability across services, suggesting that while some services continued to experience elevated demand, others are seeing a return toward pre-pandemic referral levels.

The analysis of three services with data from pre- to post-pandemic periods showed *a* ~ 51% increase in monthly referrals during the pandemic, which remained ~31% higher than the pre-pandemic baseline in the post-pandemic period. Data from all services indicated an ~8% decline in monthly FREED referrals in the post-pandemic period compared to during the pandemic, which may suggest that referrals have declined slightly post-pandemic but remain elevated overall (vs. unobserved pre-pandemic baselines). These findings align with trends reported both in the UK and internationally. For example, in Denmark, a temporary increase in diagnosed EDs among youth during the pandemic was followed by a return to pre-pandemic levels in most age groups except emerging adults, where increases persisted [10]. Similar trends have been observed in Germany and the US with sustained increases in hospitalizations for EDs post-pandemic [8, 9]. Together, these findings suggest that pandemic-related disruptions may have led to lasting changes in the incidence of EDs in emerging adults. Consistent trends across all services were a decrease in the proportion of AN diagnoses and an increase in average DUED in the post-pandemic period compared to during the pandemic. Of note, and consistent with our earlier findings, there were no changes in BMI for AN patients or symptom severity across all patients across the pandemic periods.

In contrast with our earlier study [20], data from all services now show an increase in average DUED of referrals in the post-pandemic period, compared to pre- or mid- pandemic. A substantial portion of DUED data is missing in the post-pandemic period (53.12%), likely in part due to delays in data collection (e.g., DUED not being determined until assessment). Prior research shows shorter mean DUED for AN presentations relative to other ED diagnoses [27, 28], so increasing DUED is consistent with the shift away from AN to a greater diagnostic mix in the post-pandemic period. Additionally, an increase in average DUED may also reflect help-seeking delays due to restrictions, fear of infection, or disruptions in healthcare access. Delayed-help seeking was widely reported across mental health services during COVID-19 [29]. Additionally, pandemic-related challenges – such as disrupted routines, isolation, weakened support networks, and weight-stigmatizing messages – likely compounded difficulties for young people needing ED treatment [30–32]. Such pandemic-related disruptions to treatment and service access likely compromised early intervention efforts for young people.

Assessment and treatment waiting times varied between FREED services. While treatment waiting times are descriptively showing signs of improvement in the post-pandemic period, they have not yet statistically returned to pre-pandemic levels in the UK. These findings align with evidence from children and young people’s ED services, which suggest that treatment waiting times are beginning to recover toward the end of 2023 [33].

This study had an exclusive focus on first-episode recent-onset illnesses (DUED ≤3 years), which offers valuable insights into this patient group, but means the findings do not extend to individuals with longer illness durations. Additionally, this research addresses a critical gap by contributing to the limited body of evidence examining post-pandemic trends in ED services. However, given that certain demographic groups experience prolonged delays in help-seeking and accessing services (e.g., [34–36]), this analysis is limited to those with EDs who actually present to a specialist treatment service. This could potentially exclude the experiences of individuals who do not or cannot seek such care. Furthermore, while the national FREED dataset shares the common limitations of real-world clinical datasets – being flawed, uncertain, proximate, and sparse [37] – it encompasses a large sample of patients, making it a valuable resource for identifying trends in young people with newly or recently diagnosed EDs.

While some indicators have returned to pre-pandemic levels, others highlight sustained challenges with potential implications for clinician wellbeing and workforce sustainability. Qualitative evidence from FREED clinicians during the pandemic described difficulties such as changes in working practices, reduced team communication, and challenges conducting online therapy [38]. Similarly, other studies reported impacts on wellbeing, including isolation, decreased confidence in tele-working [39], and concerns about the efficacy of therapeutic interventions [40]. Monitoring these trends will be essential to support both clinicians and services in meeting the ongoing needs of young people with EDs.

Building on the existing literature exploring the impact of COVID-19 on ED services remains essential. In our earlier paper, we concluded the need for investment in ED to align with the increased referral trends observed during the pandemic. The present findings reinforce this recommendation, as referrals have not returned to pre-pandemic levels and these referrals are not less severe in their ED presentation, as also found previously [20]. Moreover, ED services have historically been, and remain, critically underfunded [41], with significant resource gaps. ED research is also underfunded compared to other areas of mental and physical health, despite the high prevalence and severity of these disorders [42, 43, 16, 44]. These challenges threaten the sustainability and effectiveness of early intervention services. Close and continued evaluation is essential to guide evidence-based ED policy development on early intervention in the UK and elsewhere.

In conclusion, this study explores the complex and lasting impact of the COVID-19 pandemic on presentations to care for young people with recent-onset EDs accessing early intervention services. Findings show that early intervention cannot yet be considered business-as-usual and referrals to ED services remain elevated post-pandemic. Sustainable financial investment and nationally driven support are required to sustain and improve the operation of FREED across England.

## Supporting information

10.1192/j.eurpsy.2025.10038.sm001 Gallagher et al. supplementary material Gallagher et al. supplementary material

## Data Availability

The data used in this study is not publicly available.

## Supplementary material

The supplementary material for this article can be found at http://doi.org/10.1192/j.eurpsy.2025.10038.
