# Supplementary material for: The continuing impact of COVID-19 on eating disorder early intervention services in England: An investigation of referral numbers and presentation characteristics
Source: Eur Psychiatry. 2025 May 30;68(1):e67. doi: 10.1192/j.eurpsy.2025.10038 (PMC12188348; doi:10.1192/j.eurpsy.2025.10038)
Supplement: Gallagher et al. supplementary material [file S0924933825100382sup001.docx]

**Supplementary Material**

Appendix 1: Wait Times Analysis

Appendix 2: Variance Explained by Linear Mixed Models (LMMs)

**Appendix 1: Wait Times Analysis**

Supplementary table 1 shows the mean number of days patients waited to be offered assessment and treatment appointments across the three pandemic periods.

**Supplementary Table 1**

|  | **Pre-pandemic** | **During pandemic** | **Post-pandemic** |
| --- | --- | --- | --- |
| **Mean wait time for offered assessment (days) (SD)** | 23.56 (18.62) | 27.93 (40.89) | 20.53 (31.53) |
| **Mean wait time for offered treatment (days) (SD)** | 66.76 (50.02) | 87.80 (92.6) | 66.43 (67.00) |

Figure S1 shows the average number of days patients waited to be offered assessment and treatment appointments for the three services with complete data spanning the three pandemic periods. Assessment waiting times increased significantly during the pandemic period, with an estimated increase of 40% compared to the pre-pandemic period (exp(β) = 1.40, SE = 0.12, t(165.17) = 2.80, p <0.05). There was no significant difference in assessment wait times between the pre-pandemic and post-pandemic period (exp(β) = 1.15, SE = 0.11, z = 1.30, p = .20).

Treatment waiting times also increased significantly during the pandemic period, with an estimated increase of 49% compared to the pre-pandemic period (exp(β) = 1.49, SE = 0.10, t(151.07) = 3.85, p < 0.001). While treatment times appear to decrease descriptively in the post-pandemic period, the LMM estimates that treatment wait times remain 72% higher in the post-pandemic period compared to the pre-pandemic period (exp(β) = 1.72, SE = 0.10, t(151.03) = 5.64, p < 0.001).

**S1**

*Average wait times to offered assessment and treatment for three services providing First Episode Rapid Early Intervention for Eating Disorders (FREED) from the pre-pandemic to post-pandemic period (January 2019-September 2024).*

Figure S2 shows the shows the average number of days patients waited to be offered assessment and treatment appointments for all services. There was no significant difference in assessment wait times between the pandemic and post-pandemic period (exp(β) = 0.93, SE = 0.04, t(1660.49) = -1.69, p = 0.09).

Similarly, there was no significant difference in treatment wait times between the pandemic and post-pandemic period (exp(β) = 1.07, SE = 0.04, t, t(1396.00) = 1.60, p = 0.10). However, descriptively, the wait for treatment appears to be decreasing in the post-pandemic period.

**S2**

*Average wait times to offered assessment and treatment for all services providing First Episode Rapid Early Intervention for Eating Disorders (FREED) from the pandemic to post-pandemic period (March 2020 to September 2024).*

**Appendix 2: Variance Explained by Linear Mixed Models (LMMs)**

Supplementary table 2 presents the percentage of variance explained for each outcome variable in models where the LMM was statistically significant. The variance explained accounts for both fixed effects and random effects.

**Supplementary Table 2**

|  | **Sample 1** | **Sample 2** |
| --- | --- | --- |
| **LMM Outcome** | **Variance Explained (%)** | |
| **Logged monthly referrals** | 66.8 | 57.9 |
| **AN diagnoses (%)** | 68.0 | 83.3 |
| **Logged DUED** | 8.1 | 10.2 |
| **Logged assessment wait times** | 55.8 | 80.3 |
| **Logged treatment wait times** | 47.2 | 55.3 |
